# The Neutrophil Percentage-to-Albumin Ratio as a New Predictor of All-Cause Mortality in Patients with Cardiogenic Shock

**DOI:** 10.1155/2020/7458451

**Published:** 2020-11-26

**Authors:** Yue Yu, Yu Liu, Xinyu Ling, Renhong Huang, Suyu Wang, Jie Min, Jian Xiao, Yufeng Zhang, Zhinong Wang

**Affiliations:** ^1^Department of Cardiothoracic Surgery, Changzheng Hospital, Naval Medical University, Shanghai 200003, China; ^2^Department of Gastroenterology, Jinling Hospital, No. 305 Zhongshan East Road, Nanjing, 210002 Jiangsu, China; ^3^Department of General Surgery, Changzheng Hospital, Naval Medical University, Shanghai 200003, China; ^4^Bethune International Peace Hospital, Shijiazhuang 050082, China

## Abstract

**Background:**

Although the neutrophil percentage-to-albumin ratio (NPAR) has proven to be a robust systemic inflammation-based predictor of mortality in a wide range of diseases, the prognostic value of the NPAR in critically ill patients with cardiogenic shock (CS) remains unknown. This study aimed at investigating the association between the admission NPAR and clinical outcomes in CS patients using real-world data.

**Methods:**

Critically ill patients diagnosed with CS in the Medical Information Mart for Intensive Care-III (MIMIC-III) database were included in our study. The study endpoints included all-cause in-hospital, 30-day, and 365-day mortality in CS patients. First, the NPAR was analyzed as a continuous variable using restricted cubic spline Cox regression models. Second, X-tile analysis was used to calculate the optimal cut-off values for the NPAR and divide the cohort into three NPAR groups. Moreover, multivariable Cox regression analyses were used to assess the association of the NPAR groups with mortality.

**Results:**

A total of 891 patients hospitalized with CS were enrolled in this study. A nonlinear relationship between the NPAR and in-hospital and 30-day mortality was observed (all *P* values for nonlinear trend<0.001). According to the optimal cut-off values by X-tile, NPARs were divided into three groups: group I (NPAR < 25.3), group II (25.3 ≤ NPAR < 34.8), and group III (34.8 ≤ NPAR). Multivariable Cox analysis showed that higher NPAR was independently associated with increased risk of in-hospital mortality (group III vs. group I: hazard ratio [HR] 2.60, 95% confidence interval [CI] 1.72-3.92, *P* < 0.001), 30-day mortality (group III vs. group I: HR 2.42, 95% CI 1.65-3.54, *P* < 0.001), and 365-day mortality (group III vs. group I: HR 6.80, 95% CI 4.10-11.26, *P* < 0.001) in patients with CS.

**Conclusions:**

Admission NPAR was independently associated with in-hospital, 30-day, and 365-day mortality in critically ill patients with CS.

## 1. Introduction

Cardiogenic shock (CS) is a high-acuity and hemodynamically diverse state of the heart pump failure characterized by a low cardiac output (CO) leading to life-threatening end-organ hypoperfusion and hypoxia [1, 2]. The most common cause of CS is myocardial dysfunction in the setting of acute myocardial infarction (AMI) [1, 3]. The all-cause in-hospital mortality (between 27% and 51%) for CS remains unacceptably high despite advances in reperfusion strategies and inotropic support during the last two decades [4–6]. Given the poor prognosis of critically ill patients with CS, it is necessary to find an accurate yet user friendly prognostic predictor for risk stratification to provide more accurate prognostic information and help implement appropriate treatment.

CS involves a complex physiological process caused by a profound depression of myocardial contractility, and its pathogenesis has not been fully elucidated. There is mounting evidence that the development of systemic inflammatory response syndrome (SIRS) plays an important role in both the pathogenesis of shock and the adverse outcomes of CS patients [7–10]. Determining peripheral leukocyte, predominantly neutrophils, and count is an inexpensive and widely available way to assess the presence of any inflammation. High levels of neutrophils in ST-elevation myocardial infarction (STEMI) patients have been found to be independently associated with an increased risk of developing late CS [11]. Albumin is a medium-sized protein that accounts for more than half of the whole serum body's composition with a molecular weight of 66-69 kDa [12]. It is established that albumin has several functions including osmotic pressure regulation and antioxidant and anti-inflammatory effects [13, 14]. One recent study showed that hypoalbuminemia was a frequent finding early in CS and was associated with mortality independent of other risk factors [15]. Recently, several studies have combined these two markers and found that the neutrophil percentage-to-albumin ratio (NPAR) could serve as an inflammation-based prognostic predictor in patients with STEMI [16], acute kidney injury [17], septic shock [18], rectal cancer [19], or palliative pancreatic cancer [20]. Nevertheless, to the best of our knowledge, no previous study has explored the prognostic value of NPARs in critically ill patients with CS. The purpose of this study was to investigate the association between the admission NPAR level and mortality in critically ill patients with CS.

## 2. Methods

### 2.1. Data Source and Ethical Statement

All the relevant data were collected from the Medical Information Mart for Intensive Care-III (MIMIC-III) database. MIMIC-III is a freely accessible critical care database covering over 50,000 hospital admissions comprised of 38,645 adults as well as 7,875 neonates admitted to surgical, trauma surgery, coronary, and cardiac surgery recovery intensive care units (ICUs) of the Beth Israel Deaconess Medical Center (BIDMC) in Boston from 2001 to 2012 [21, 22]. The MIMIC-III database documents included charted events such as demographic data, vital signs, laboratory findings and blood gas analysis data, scoring systems, and survival outcomes. We passed the “Protecting Human Research Participants” exam and obtained permission to access the dataset (authorization code: 33281932). The establishment of the MIMIC-III database is approved by the Institutional Review Boards (IRB) of the Massachusetts Institute of Technology (MIT, Cambridge, MA, USA) and BIDMC. Our study utilized the anonymous data available from this database, and hence, the requirement for informed consent was waived. In summary, the study was complied with the ethical standards laid down in the 1964 Declaration of Helsinki and its later amendments. In addition, we conducted this study in accordance with the STrengthening the Reporting of OBservational studies in Epidemiology (STROBE) statement [23].

### 2.2. Population Selection

We included all ICU patients (aged >18 years) with the primary diagnosis of CS using International Classification of Diseases (ICD)-9 diagnosis codes (ICD-9 codes for CS: 785.51 and 998.01) in the MIMIC-III database. Patients were excluded if they had [1] multiple admissions other than the first admission; [2] a secondary diagnosis of cancer, anemia, hematologic diseases, myelodysplastic syndrome, and liver disease on admission; [3] a length of stay in the ICU less than 24 hours; or [4] incomplete or unobtainable documented neutrophil percentage, albumin, or other important data records.

### 2.3. Data Extraction, Processing, and Definitions

The data were extracted from the database using the structured query language (SQL) with PostgreSQL (version 9.4.6, http://www.postgresql.org). The code that supports the MIMIC-III documentation and website is publicly available, and contributions from the community of users are encouraged (https://github.com/MIT-LCP/mimic-website). Demographic information included age, sex, smoking, and body mass index (BMI). BMI was calculated as weight (kg) divided by height squared (m^2^), using the height and weight reported at the time of admission. History of disease included chronic heart failure (CHF), atrial fibrillation (AF), coronary artery disease (CAD), chronic obstructive pulmonary disease (COPD), hypertension, stroke, diabetes mellitus (DM), and chronic kidney disease (CKD). Vital signs on admission included systolic blood pressure (SBP), diastolic blood pressure (DBP), heart rate (HR), and pulse oximetry-derived oxygen saturation (SpO_2_). Laboratory findings and blood gas analysis data included neutrophil percentage, albumin, NPAR, creatinine, glucose, blood urea nitrogen (BUN), hemoglobin, blood platelet count, white blood cell (WBC) count, cardiac troponin t (cTnT), bicarbonate, potassium, sodium, chloride, lactate, anion gap, activated partial thromboplastin time (APTT), prothrombin time (PT), and international normalized ratio (INR). If patients received a laboratory test more than once during their hospitalization, only the initial test results were included for analysis. Three scoring systems (the Oxford Acute Severity of Illness Score [OASIS], the Sequential Organ Failure Assessment [SOFA], and the Simplified Acute Physiology Score II [SAPS II]) were calculated within the first 24 hours after admission using the values associated with the greatest severity of illness. Treatment information data included oxygen therapy (noninvasive ventilation [NIV] or invasive ventilation during hospitalization), percutaneous coronary intervention (PCI), coronary artery bypass grafting (CABG), intraaortic balloon pump (IABP), renal replacement treatment (RRT), and in-hospital medication (inotrope and vasoconstrictor) administration.

The neutrophil percentage was defined as the percentage of neutrophils in white blood cells. The NPAR was calculated as the neutrophil percentage as the numerator divided by albumin using the same blood samples drawn on admission according to the formula:  (Neutrophil percentage (%)∗100/Albumin (g/dL)).

### 2.4. Endpoints

The primary endpoint of our study was all-cause in-hospital mortality, which was defined as the survival status at hospital discharge. We selected all-cause 30-day and 365-day mortality as secondary endpoints. Patients with missing survival outcome information were excluded from the final cohort.

### 2.5. Statistical Analysis

Baseline characteristics of enrolled participants were presented by using either Student's *t*-test, Kruskal Wallis rank test, Pearson's *χ*^2^ test or Fisher's exact test as appropriate. Continuous variables were characterized as mean (standardized differences [SD]) or median (interquartile range [IQR]), while categorical or ranked data were presented as count and proportion.

Restricted cubic spline Cox regression models were used to evaluate the possible nonlinear relationship between the NPAR and mortality [24]. If the test for nonlinearity was not significant, the test result for overall association and linearity was checked, with significant results indicating linear associations.

X-tile software (version 3.6.1; Yale University, New Haven, CT, USA) based on the maximal log-rank chi-square value was applied to determine the optimal cut-off values of NPARs [25–29]. In our study, NPARs were divided into three groups: group I (NPAR < 25.3), group II (25.3 ≤ NPAR < 34.8), and group III (34.8 ≤ NPAR). The Kaplan-Meier (KM) method was used to plot unadjusted survival curves, and the log-rank test was used to compare differences between the three NPAR groups. Multivariable Cox regression analysis was used to estimate the hazard ratios (HRs) and their corresponding 95% confidence intervals (CIs) of mortality and adjust for the confounding variables that were selected based on *P* ≤ 0.05 in the univariable analysis. The Akaike information criterion (AIC) was applied as the selection criterion for the optimal model. In addition, receiver operating characteristic (ROC) curve analysis was performed to compare the sensitivity and specificity of the NPAR with those of the neutrophil percentage and albumin. Furthermore, interaction and subgroup analyses were performed to investigate the association between the NPAR and in-hospital mortality according to age, sex, smoking status, etiology, scoring systems, and treatment strategies.

As extensive missing data might lead to bias, variables with over 20% missing values were not included in the subsequent analyses. Correspondingly, multivariate imputation (MI) was used for variables with less than 20% missing values [30, 31].

A two-tailed *P* value of less than 0.050 was considered to be statistically significant. All statistical analyses were performed using SPSS software (version 22.0; IBM Corporation, St. Louis, Missouri, USA) and R software (version.3.6.1; The R Project for Statistical Computing, TX, USA; http://www.r-project.org).

## 3. Results

### 3.1. Subject and Variable Characteristics

**Figure 1:**
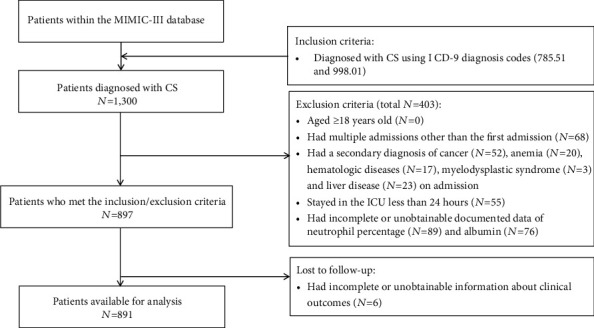
Flow chart of cohort selection. Abbreviation: MIMIC-III: Medical Information Mart for Intensive Care-III; CS: cardiogenic shock; ICD: international classification of diseases; ICU: intensive care unit.

After application of the inclusion and exclusion criteria, the final study cohort consisted of 891 CS patients (Figure 1). The median age was 71.8 (61.6-80.8) years, while 59.7% (532/891) subjects were male. The comparison of baseline characteristics between the three NPAR groups is summarized in Table 1. 36.5% (325/891) patients were in the low NPAR group (group I: NPAR < 25.3), 44.6% (397/891) patients were in the mid NPAR group (group II: 25.3 ≤ NPAR < 34.8), and 19.0 (169/891) patients were in the high NPAR group (group III: 34.8 ≤ NPAR). Patients in the highest NPAR group were older (*P* = 0.004) and had higher prevalence of AF (*P* = 0.030) and COPD (*P* = 0.031). They had higher laboratory findings and severity scores (neutrophil percentage: *P* < 0.001; BUN: *P* = 0.024; creatinine: *P* = 0.043; lactate: *P* = 0.010; anion gap: *P* = 0.027; APTT: *P* = 0.002; PT: *P* < 0.001; INR: *P* < 0.001; SOAF: *P* < 0.001, OASIS: *P* < 0.001, SAPS II: *P* < 0.001). Furthermore, patients in the highest NPAR group tended to receive oxygen therapy (*P* < 0.001) and in-hospital medication (vasoconstrictor: *P* = 0.002).

### 3.2. Relationship between the NPAR and Mortality

**Figure 2:**
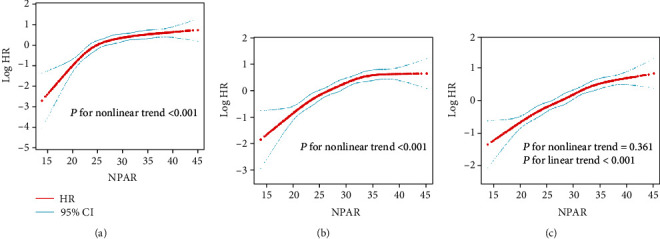
Association of the admission NPAR level with mortality in restricted cubic spline models. (a) in-hospital mortality. (b) 30-day mortality. (c) 365-day mortality. The red and blue lines represent the estimated log HR and the 95% CI, respectively. Abbreviation: NPAR: neutrophil percentage-albumin ratio; HR: hazard ratio; CI: confidence interval.

Restricted cubic spline analyses showed the nonlinear relationships of NPAR and risk of all-cause in-hospital and 30-day mortality. (all *P* values for nonlinear trend <0.001; Figures 2(a) and 2(b)). However, a linear relationship between the NPAR and one-year mortality could be observed (*P* for nonlinear trend=0.361; *P* for linear trend <0.001; Figure 2(c)).

### 3.3. Survival Analysis

**Figure 3:**
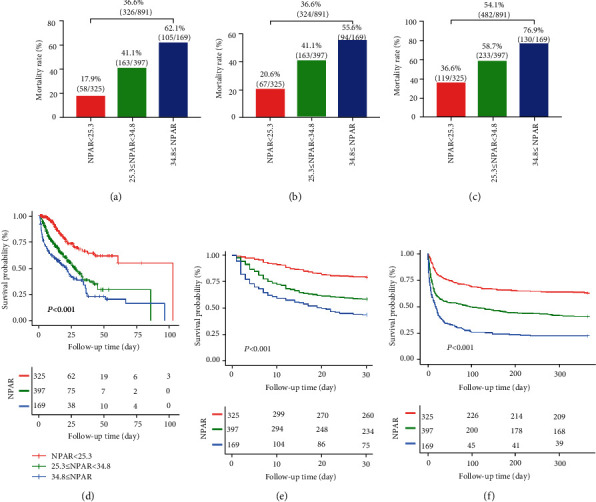
Mortality rate and Kaplan–Meier curves of three NPAR groups. (a, d) in-hospital mortality. (b, e) 30-day mortality. (c, f) 365-day mortality. Abbreviation: NPAR: neutrophil percentage-albumin ratio.

Among the 891 CS patients included, 36.6% (326/891) died in the hospital, 36.4% (324/891) died during the first 30 days, and 54.1% (482/891) died during the one-year follow-up period (Figure 3). Kaplan-Meier curves for the low, intermediate, and high RDW groups showed that a higher RDW value was significantly associated with an enhanced risk of all-cause mortality (log-rank test: *P* < 0.001 for all clinical outcomes) (Figure 3). Cox regression models were applied to determine the association between the different NPAR groups and clinical outcomes among patients with CS. Group I was considered as the reference group. In the univariable Cox regression analysis, group III and group II were associated with an increased risk of all-cause mortality compared to group I (Table 2). In the multivariable Cox regression models, we adjusted for potential covariates with *P* ≤ 0.05 in the univariable analysis (Table S1-3). In the multivariable analysis, a higher NPAR value was identified as an independent predictor of all-cause in-hospital mortality (group II vs. group I: HR 2.22, 95% CI 1.55-3.16, *P* < 0.001; group III vs. group I: HR 2.60, 95% CI 1.72-3.92, *P* < 0.001), 30-day mortality (group II vs. group I: HR 1.96, 95% CI 1.42-2.71, *P* < 0.001; group III vs. group I: HR 2.42, 95% CI 1.65-3.54, *P* < 0.001), and 365-day mortality (group II vs. group I: HR 3.03, 95% CI 2.14-4.28, *P* < 0.001; group III vs. group I: HR 6.80, 95% CI 4.10-11.26, *P* < 0.001) in critically ill patients with CS (Table 2).

### 3.4. Comparison between Neutrophil Percentage, Albumin, and the NPAR

The ROC curves were generated, and we found that the AUCs of in-hospital mortality for the NPAR, neutrophil percentage, and albumin were 0.69 (95% CI 0.66-0.73), 0.56 (95% CI 0.53-0.61), and 0.57 (95% CI 0.53-0.61), respectively (Figure S1 and Table 3). Comparing AUCs, the NPAR was found to be a better predictor than the neutrophil percentage or albumin alone (*P* < 0.001). The results of in-hospital mortality were consistent with the results for 30-day and 365-day mortality. In the Cox regression analysis, the neutrophil percentage (HR 2.09, 95% CI 1.59-2.75, *P* < 0.001) and albumin (HR 0.98, 95% CI 0.97-0.99, *P* < 0.001) were independently associated with 365-day mortality; they did not remain statistical significance in the multivariable Cox regression models for in-hospital and 30-day mortality (Table S1-3). The NPAR remains an independent indicator in all the multivariable Cox regression models (Table 2 and S1-3).

### 3.5. Sensitivity and Subgroup Analyses

A series of sensitivity analyses were performed to validate the robustness of our findings. Subgroup analysis showed higher NPAR values that were also associated with deteriorative mortality in most strata except in patients with a medical history of hypertension and receiving PCI or RRT (Table 4).

## 4. Discussion

Our main findings can be summarized as follows. First, a nonlinear relationship between the admission NPAR and in-hospital and 30-day mortality could be observed. Second, a higher NPAR was associated with increased risk of in-hospital, 30-day, and 365-day mortality in CS patients. Third, after adjustments for potential confounders, admission NPAR was identified as an independent predictor of clinical outcomes in CS patients. Fourth, the NPAR was proven to be a better predictor of outcomes than either albumin or the neutrophil percentage alone. To the best of our knowledge, this study is the first to investigate the prognostic value of NPAR in critically ill patients with CS.

Our findings were consistent with the results of studies that evaluated the prognostic value of NPARs in other clinical settings including STEMI [16], acute kidney injury [17], septic shock [18], rectal cancer [19], and palliative pancreatic cancer [20]. Additionally, several studies have investigated other measurable laboratory markers related to the inflammatory response in CS. Sionis et al. found increased levels of leukocytes and platelet-derived circulating microparticles (cMPs) in STEMI patients complicated by CS [32]. Similar reports by Barron et al. suggested that there was an association between high leukocyte count and the incidence of CS or congestive HF after myocardial infarction [33]. In addition, Yost et al. showed that an increased preoperative neutrophil-to-lymphocyte ratio (NLR) was associated with worse outcomes in CS patients requiring extracorporeal membrane oxygenation (ECMO) [34]. In addition, one recent study found that a high-sensitivity C-reactive protein (CRP) to the albumin ratio was independently correlated with short-term major adverse cardiac events including CS in patients with acute coronary syndrome (ACS) [35]. Furthermore, preimplantation hypoalbuminemia was identified as a strong factor associated with mortality in patients undergoing ECMO instituted for CS [36]. However, most of these previous studies only focused on CS patients in the setting of a STEMI. Although the most common cardiac cause of CS is AMI, CS can also result from nonischemic cardiac conditions [37]. In the subgroup analysis of our study, we found that the NPAR was a significant predictor of poor prognosis in CS of different etiologies. Consequently, we hope the results of this study will supplement the findings of previous studies. In addition, most previous studies used medians or quartiles as cut-off points of their prognostic predictors. In this study, X-tile software, a bioinformatics statistical tool for biomarker assessment, was applied to determine the optimal outcome-based cut-off values of NPAR. Furthermore, we also analyzed the NPAR as a continuous variable using restricted cubic splines and showed the nonlinear relationship between the NPAR and short-term clinical outcomes in CS patients.

CS involves a complex physiological process, which is the result of acute to subacute derangements in the entire circulatory system [10]. CS has traditionally been seen as mainly a mechanical disease where impairment of myocardium and reduction in myocardial contractility lead to a vicious spiral of reduction in cardiac output and blood pressure and ultimately multiple organ failure (MOF) and death. However, new evidence has suggested that SIRS, expression of endothelial (eNOS) and inducible nitric oxide synthase (iNOS), and release of inflammatory cytokines occur frequently with increasing durations of CS [38]. High levels of NO and peroxynitrite produced by eNOS and iNOS have cardiac toxicity, and some inflammatory markers lead to inappropriate vasodilatation and decreased systemic vascular resistance (SVR) [39]. Additionally, there is an increase in neutrophils and monocytes and a simultaneous reduction in lymphocytes and eosinophils with leukocyte activation during SIRS [40]. Neutrophils intermediate the early inflammatory response, and they were found to be most useful among all leukocyte subsets for predicting cardiovascular mortality [41]. Furthermore, acute and chronic inflammatory conditions could affect serum albumin levels by altering hepatic protein metabolism and inducing capillary leakage [42]. Most of the existing evidence has suggested that a low albumin level is not an appropriate parameter rather than reflects poor nutritional status but rather the severity of inflammation and illness in acute disease [43, 44]. Therefore, it appears that the combination of neutrophils and albumin could serve as an acute inflammatory response marker in CS patients.

Even in the era of reperfusion therapy, CS remains one of the leading causes of death with in-hospital mortality rates still approaching 50% [45, 46]. Individualized and timely risk assessment for each critically ill patient allows a more precise decision-making for therapeutic strategy and medical resource allocation. Both neutrophil percentage and albumin tests are rapid, easy, and inexpensive laboratory tests that could provide information about the status of the patient's blood contents. The combination of the neutrophil percentage and albumin provides a fast evaluation of risks for patients with CS. Furthermore, even under conditions without imaging or additional laboratory tests, the NPAR could still serve as an effective marker for quick risk assessments provided a complete blood count and biochemistry test that can be performed. Our findings might provide additional convenience in some special situations, for example, underdeveloped areas.

Several limitations of our study should be noted. First, the study was a single-center retrospective design and was therefore subject to selection bias. Second, we extracted the NPAR data in patients only upon admission to the ICU and did not assess changes before and during the ICU stay. Third, although we have done our best to use a multivariate model to control for bias, there remain numerous other known and unknown factors that might confound the results. Fourth, we did not include detailed information with regard to some important clinical or laboratory variables (such as left ventricular ejection fraction [LVEF] and brain natriuretic peptide [BNP]) due to more than 20% missing values. Furthermore, considering that the ROC value of NPAR was not satisfying, it was necessary to develop a multivariable model or scoring system, with the inclusion of NPAR, to better predict the clinical outcomes of CS patients.

## 5. Conclusions

A higher NPAR on admission was associated with an increased risk of all-cause in-hospital, 30-day, and 365-day mortality in critically ill patients with CS.

## Figures and Tables

**Table 1:** Baseline characteristics of patients with CS between three NPAR groups.

Characteristics	Total	Group I (NPAR < 25.3)	Group II (25.3 ≤ NPAR < 34.8)	Group III (34.8 ≤ NPAR)	*P* value
Number	891	325	397	169	
Demographics					
Age, years	71.8 (61.6-80.8)	68.2 (58.9-80.2)	73.0 (62.9-81.2)	74.1 (65.1-80.6)	0.004
Sex, male	532 (59.7%)	182 (56.0%)	250 (63.0%)	100 (59.2%)	0.162
Smoking	463 (52.0%)	165 (50.8%)	218 (54.9%)	80 (47.3%)	0.221
BMI, kg/m^2^	27.6 (23.8-31.6)	27.7 (23.8-31.8)	27.7 (23.6-31.8)	27.5 (23.9-30.2)	0.618
Etiology					<0.001
AMI	671 (75.4%)	215 (66.2%)	312 (78.6%)	144 (85.2%)	
AHF	179 (20.1%)	89 (27.4%)	73 (18.4%)	17 (10.1%)	
Others/unknown	41 (4.5%)	21 (6.5%)	12 (3.0%)	8 (4.7%)	
History of disease					
CHF	190 (21.3%)	92 (28.3%)	79 (19.9%)	19 (11.2%)	0.002
AF	431 (48.4%)	140 (43.1%)	198 (49.9%)	93 (55.0%)	0.030
CAD	472 (53.0%)	184 (56.6%)	206 (51.9%)	82 (48.5%)	0.196
Hypertension	177 (19.9%)	64 (19.7%)	88 (22.2%)	25 (14.8%)	0.131
Stroke	26 (2.9%)	14 (4.3%)	11 (2.8%)	1 (0.6%)	0.057
COPD	23 (2.6%)	4 (1.23%)	10 (2.5%)	9 (5.3%)	0.031
DM	332 (37.3%)	126 (38.8%)	148 (37.3%)	58 (34.3%)	0.624
CKD	240 (26.9%)	81 (24.9%)	121 (30.5%)	38 (22.5%)	0.086
Vital signs at presentation					
SBP, mmHg	77.0 (67.0-86.0)	77.0 (67.0-87.0)	77.0 (68.0-86.0)	77.0 (65.0-85.0)	0.236
DBP, mmHg	46.0 (38.3-52.0)	46.0 (38.0-52.0)	46.0 (37.0-52.0)	44.0 (36.0-51.0)	0.704
HR, beats/min	99.0 (84.0-116.0)	97.0 (82.0-113.0)	101.0 (85.0-115.0)	101.0 (84.0-118.0)	0.194
SpO_2_, %	92.0 (88.0-95.0)	93.0 (88.0-95.0)	92.0 (87.0-95.0)	92.0 (88.0-95.0)	0.148
Laboratory findings and blood gas analysis					
Neutrophil percentage, %	82.8 (75.0-88.0)	75.8 (68.0-84.2)	84.0 (78.6-88.4)	87.8 (82.5-91.2)	<0.001
Albumin, mg/dL	3.0 (2.6-3.4)	3.5 (3.1-3.7)	2.900 (2.6-3.1)	2.4 (2.2-2.6)	<0.001
Creatinine, *μ*mol/L	1.3 (0.9-2.2)	1.2 (0.9-2.0)	1.4 (1.0-2.3)	1.40 (1.0-2.2)	0.043
Glucose, mg/dL	113.0 (92.0-139.5)	110.0 (91.0-135.0)	112.0 (93.0-140.0)	119.0 (92.0-143.0)	0.327
BUN, mg/dL	29.0 (19.0-47.0)	25.0 (18.0-45.0)	29.0 (20.0-50.0)	32.0 (20.0-45.0)	0.024
Hemoglobin, g/dL	9.80 (8.40-11.30)	10.300 (8.6-11.8)	9.600 (8.4-11.0)	9.5 (8.2-10.7)	0.002
Platelet, 10^9^/L	187.00 (132.0-253.0)	182.0 (136.0-243.0)	192.0 (135.0-264.0)	181.0 (126.0-242.0)	0.249
WBC, 10^9^/L	14.90 (11.10-19.50)	13.7 (10.2-18.2)	15.9 (11.9-20.2)	15.4 (12.0-19.8)	<0.001
cTnT, ng/mL	1.04 (0.12-4.67)	0.8 (0.1-3.8)	1.2 (0.2-5.0)	1.0 (0.1-5.7)	0.052
Bicarbonate, mmol/L	20.0 (17.0-23.0)	21.0 (18.0-24.0)	20.0 (17.0-23.0)	18.0 (15.0-22.0)	<0.001
Potassium, mmol/L	3.70 (3.40-4.20)	3.7 (3.4-4.1)	3.8 (3.4-4.2)	3.7 (3.3-4.3)	0.374
Sodium, mmol/L	135.00 (132.00-138.00)	135.0 (133.0-138.0)	135.0 (132.0-138.0)	135.0 (132.0-138.0)	0.635
Chloride, mmol/L	101.00 (97.00-105.00)	101.0 (98.0-105.0)	101.0 (97.0-105.0)	102.0 (98.0-106.0)	0.121
Lactate, mmol/L	3.30 (1.90-6.10)	3.0 (1.6-5.6)	3.3 (1.9-6.1)	3.8 (2.2-6.3)	0.010
Anion gap, mmol/L	14.0 (12.0-17.0)	14.0 (12.0-16.0)	14.0 (12.0-17.0)	15.0 (12.0-17.0)	0.027
APTT, second	32.7 (27.9-41.2)	31.9 (26.9-38.6)	33.2 (28.0-43.0)	34.7 (29.5-43.3)	0.002
PT, second	14.5 (13.2-17.5)	14.2 (13.0-15.9)	14.6 (13.3-17.6)	15.4 (13.5-18.9)	<0.001
INR	1.3 (1.2-1.7)	1.3 (1.1-1.5)	1.3 (1.2-1.7)	1.4 (1.2-1.8)	<0.001
Scoring system					
OASIS	37.0 (31.0-43.0)	34.0 (27.0-40.0)	38.0 (32.0-43.0)	41.0 (36.0-47.0)	<0.001
SOFA	7.0 (4.0-9.0)	6.0 (4.0-9.0)	7.0 (5.0-10.0)	8.0 (6.0-10.0)	<0.001
SAPS II	47.0 (37.0-56.0)	42.0 (32.0-52.0)	48.0 (39.0-58.0)	51.0 (44.0-59.0)	<0.001
In-hospital management					
Oxygen therapy	670 (75.2%)	217 (66.8%)	307 (77.3%)	146 (86.4%)	<0.001
PCI	502 (56.3%)	179 (55.1%)	228 (57.4%)	95 (56.2%)	0.594
CABG	119 (13.4%)	64 (19.7%)	44 (11.1%)	11 (6.5%)	<0.001
IABP	9 (1.0%)	1 (0.3%)	5 (1.3%)	3 (1.8%)	0.196
RRT	175 (19.6%)	53 (16.3%)	83 (20.9%)	39 (23.1%)	0.138
In-hospital medication					
Inotrope	352 (39.5%)	121 (37.2%)	151 (38.0%)	80 (47.3%)	0.067
Vasoconstrictor	651 (73.1%)	218 (67.1%)	296 (74.6%)	137 (81.1%)	0.003

Abbreviation: NPAR: neutrophil percentage-albumin ratio; BMI: body mass index; AMI: acute myocardial infarction; AHF: acute heart failure; CHF: chronic heart failure; AF: atrial fibrillation; CAD: coronary artery disease, COPD: chronic obstructive pulmonary disease; DM: diabetes mellitus; CKD: chronic kidney disease; SBP: systolic blood pressure; DBP: diastolic blood pressure; HR: heart rate; SpO2: pulse oximetry-derived oxygen saturation; BUN: blood urea nitrogen; WBC: white blood cell; cTnT: cardiac troponin t; APTT: activated partial thromboplastin time; PT: prothrombin time; INR: international normalized ratio; OASIS: Oxford Acute Severity of Illness Score; SOFA: Sequential Organ Failure Assessment; SAPS: Simplified Acute Physiology Score; PCI: percutaneous coronary intervention; CABG: coronary artery bypass grafting; IABP: intraaortic balloon pump; RRT: renal replacement treatment.

**Table 2:** Association between three NPAR groups and clinical outcomes in patients with CS.

	Univariable analysis		Multivariable analysis	
Clinical outcomes	HR (95% CI)	*P* value	HR (95% CI)	*P* value
In-hospital mortality				
Group I (NPAR < 25.3)	1		1	
Group II (25.3 ≤ NPAR < 34.8)	2.58 (1.90, 3.51)	<0.001	2.22 (1.55, 3.16)	<0.001
Group III (34.8 ≤ NPAR)	3.74 (2.69, 5.19)	<0.001	2.60 (1.72, 3.92)	<0.001
30-day mortality				
Group I (NPAR < 25.3)	1		1	
Group II (25.3 ≤ NPAR < 34.8)	2.34 (1.76, 3.11)	<0.001	1.96 (1.42, 2.71)	<0.001
Group III (34.8 ≤ NPAR)	3.69 (2.70, 5.05)	<0.001	2.42 (1.65, 3.54)	<0.001
365-day mortality				
Group I (NPAR < 25.3)	1		1	
Group II (25.3 ≤ NPAR < 34.8)	2.00 (1.61, 2.50)	<0.001	3.03 (2.14, 4.28)	<0.001
Group III (34.8 ≤ NPAR)	3.43 (2.67, 4.41)	<0.001	6.80 (4.10, 11.26)	<0.001

Abbreviation: CS: cardiogenic shock; NPAR: neutrophil percentage-albumin ratio; HR: hazard ratio; CI: confidence interval.

**Table 3:** Area under receiver operating characteristic curve of the neutrophil percentage, albumin, and NPAR.

Clinical outcomes	AUC	(95% CI)	*P* value
In-hospital mortality			<0.001
Neutrophil percentage	0.56	0.53-0.61	
Albumin	0.57	0.53-0.61	
NPAR	0.69	0.66-0.73	
30-day mortality			<0.001
Neutrophil percentage	0.57	0.53-0.61	
Albumin	0.54	0.50-0.58	
NPAR	0.66	0.63-0.70	
365-day mortality			<0.001
Neutrophil percentage	0.57	0.54-0.61	
Albumin	0.59	0.56-0.63	
NPAR	0.68	0.64-0.71	

Abbreviations: AUC: area under the curve; NPAR: neutrophil percentage-albumin ratio; CI: confidence interval.

**Table 4:** The association between three NPAR groups and all-cause in-hospital mortality in subgroup analysis.

		Group I	Group II		Group III		
Characteristics	No. of patients	Ref	HR (95% CI)	*P* value	HR (95% CI)	*P* value	*P* value for interaction
Age, years							0.104
≤72	450	1	2.26 (1.39, 3.70)	0.001	3.98 (2.38, 6.64)	<0.001	
>72	441	1	2.55 (1.71, 3.78)	<0.001	3.15 (2.05, 4.82)	<0.001	
Sex							0.673
Male	532	1	2.11 (1.42, 3.13)	<0.001	2.57 (1.66, 3.98)	<0.001	
Female	359	1	2.98 (1.81, 4.91)	<0.001	5.59 (3.34, 9.36)	<0.001	
Smoking							0.412
No	428	1	2.91 (1.92, 4.40)	<0.001	3.01 (1.93, 4.70)	<0.001	
Yes	463	1	2.33 (1.47, 3.69)	<0.001	5.01 (3.07, 8.17)	<0.001	
Etiology							0.263
AMI	671	1	3.62 (1.31, 10.01)	0.013	9.10 (3.37, 24.59)	<0.001	
AHF	179	1	2.67 (1.30, 5.48)	0.008	2.81 (1.72, 5.86)	0.004	
CHF							0.188
No	701	1	2.51 (1.75, 3.61)	<0.001	4.67 (3.16, 6.89)	<0.001	
Yes	190	1	2.64 (1.48, 4.73)	0.001	2.80 (1.53, 5.13)	0.001	
AF							0.215
No	460	1	2.09 (1.36, 3.22)	0.001	3.49 (2.20, 5.56)	<0.001	
Yes	431	1	3.16 (2.03, 4.93)	<0.001	4.03 (2.52, 6.45)	<0.001	
CAD							0.305
No	419	1	3.05 (1.94, 4.80)	<0.001	4.52 (2.82, 7.26)	<0.001	
Yes	472	1	2.13 (1.40, 3.24)	<0.001	3.11 (1.96, 4.95)	<0.001	
Hypertension							0.502
No	714	1	2.96 (2.06, 4.25)	<0.001	4.30 (2.95, 6.27)	<0.001	
Yes	177	1	1.57 (0.87, 2.82)	0.131	2.38 (1.17, 4.85)	0.017	
DM							0.890
No	559	1	2.59 (1.77, 3.79)	<0.001	3.22 (2.15, 4.82)	<0.001	
Yes	332	1	2.70 (1.59, 4.59)	<0.001	5.27 (2.98, 9.33)	<0.001	
CKD							0.807
No	651	1	2.95 (2.01, 4.34)	<0.001	4.44 (2.98, 6.63)	<0.001	
Yes	240	1	1.90 (1.14, 3.17)	0.013	2.45 (1.34, 4.47)	0.004	
OASIS							0.785
≤37	468	1	2.50 (1.61, 3.90)	<0.001	3.43 (2.06, 5.71)	<0.001	
>37	423	1	2.18 (1.41, 3.35)	<0.001	2.96 (1.89, 4.64)	<0.001	
SOFA							0.978
≤ 7	491	1	2.68 (1.73, 4.14)	<0.001	3.81 (2.34, 6.18)	<0.001	
>7	400	1	2.24 (1.45, 3.47)	<0.001	3.16 (2.00, 4.99)	<0.001	
SAPS II							0.303
≤ 47	464	1	3.46 (2.10, 5.71)	<0.001	5.14 (2.95, 8.97)	<0.001	
>47	427	1	1.75 (1.19, 2.59)	0.005	2.34 (1.55, 3.52)	<0.001	
Oxygen therapy							0.852
No	221	1	2.24 (1.20, 4.17)	0.011	3.39 (1.58, 7.31)	0.002	
Yes	670	1	2.59 (1.82, 3.70)	<0.001	3.65 (2.51, 5.29)	<0.001	
PCI							0.413
No	389	1	2.97 (2.10, 4.21)	<0.001	4.47 (3.08, 6.48)	<0.001	
Yes	502	1	1.43 (0.74, 2.74)	0.288	2.17 (1.08, 4.38)	0.030	
RRT							0.131
No	716	1	3.00 (2.08, 4.34)	<0.001	4.84 (3.27, 7.16)	<0.001	
Yes	175	1	1.70 (0.97, 2.97)	0.062	1.84 (0.99, 3.41)	0.054	
Inotrope							0.164
No	539	1	2.13 (1.47, 3.09)	<0.001	3.05 (2.02, 4.60)	<0.001	
Yes	352	1	3.57 (2.06, 6.16)	<0.001	5.34 (3.02, 9.44)	<0.001	
Vasoconstrictor							0.485
No	240	1	2.05 (1.17, 3.62)	0.013	2.58 (1.34, 4.97)	0.005	
Yes	651	1	2.78 (1.92, 4.02)	<0.001	4.13 (2.80, 6.11)	<0.001	

Abbreviation: NPAR: neutrophil percentage-albumin ratio; HR: hazard ratio; CI: confidence interval; BMI: body mass index; AMI: acute myocardial infarction; AHF: acute heart failure; CHF: chronic heart failure; AF: atrial fibrillation; CAD: coronary artery disease; DM: diabetes mellitus; CKD: chronic kidney disease; OASIS: Oxford Acute Severity of Illness Score; SOFA: Sequential Organ Failure Assessment; SAPS: Simplified Acute Physiology Score; PCI: percutaneous coronary intervention; RRT: renal replacement treatment.

## Data Availability

The clinical data used to support the findings of this study were supplied by the Monitoring in Intensive Care Database III version 1.4 (MIMIC-III v.1.4). Although the database is publicly and freely available, researchers must complete the National Institutes of Health's web-based course known as Protecting Human Research Participants to apply for permission to access the database.

## Abbreviations

CS: Cardiogenic shock
CO: Cardiac output
AMI: Acute myocardial infarction
SIRS: Systemic inflammatory response syndrome
STEMI: ST-elevation myocardial infarction
NPAR: Neutrophil percentage-to-albumin ratio
MIMIC-III: Medical Information Mart for Intensive Care-III
ICU: Intensive care unit
BIDMC: Beth Israel Deaconess Medical Center
IRB: Institutional review boards
MIT: Massachusetts institute of technology
STROBE: STrengthening the Reporting of OBservational studies in Epidemiology
ICD: International classification of diseases
SQL: Structured query language
BMI: Body mass index
CHF: Chronic heart failure
AF: Atrial fibrillation
CAD: Coronary artery disease
COPD: Chronic obstructive pulmonary disease
DM: Diabetes mellitus
CKD: Chronic kidney disease
SBP: Systolic blood pressure
DBP: Diastolic blood pressure
RR: Respiratory rate
HR: Heart rate
SpO_2_: Pulse oximetry-derived oxygen saturation
BUN: Blood urea nitrogen
WBC: White blood cell
cTnT: Cardiac troponin t
APTT: Activated partial thromboplastin time
PT: Prothrombin time
INR: International normalized ratio
OASIS: Oxford Acute Severity of Illness Score
SOFA: Sequential Organ Failure Assessment
SAPS: Simplified Acute Physiology Score
NIV: Noninvasive ventilation
PCI: Percutaneous coronary intervention
CABG: Coronary artery bypass grafting
IABP: Intra-aortic balloon pump
RRT: Renal replacement treatment
SD: Standardized difference
IQR: Interquartile range
HR: Hazard ratio
CI: Confidence interval
KM: Kaplan-Meier
AIC: Akaike information criterion
ROC: Receiver-operating characteristic
MI: Multivariate imputation
cMPs: Circulating microparticles
NLR: Neutrophil to lymphocyte ratio
ECMO: Extracorporeal membrane oxygenation
CRP: C-reactive protein
ACS: Acute coronary syndrome
RDW: Red blood cell distribution width
MOF: Multiple organ failure
eNOS: Endothelial nitric oxide synthase
iNOS: Inducible nitric oxide synthase
SVR: Systemic vascular resistance
LVEF: Left ventricular ejection fraction
BNP: Brain natriuretic peptide.

## Supplementary Materials

Supplementary Materials Table S1: univariable and multivariable Cox regression analysis for all-cause in-hospital mortality. Table S2: univariable and multivariable Cox regression analysis for all-cause 30-day mortality. Table S3: univariable and multivariable Cox regression analysis for all-cause 365-day mortality. Figure S1: receiver operating characteristic curve of the neutrophil percentage, albumin, and NPAR values. (a, b) in-hospital mortality. (c, d) 30-day mortality. (e, f) 365-day mortality. Abbreviations: AUC: area under the curve; NPAR: neutrophil percentage-albumin ratio.
