# Supplementary material for: The Neutrophil Percentage-to-Albumin Ratio as a New Predictor of All-Cause Mortality in Patients with Cardiogenic Shock
Source: Biomed Res Int. 2020 Nov 26;2020:7458451. doi: 10.1155/2020/7458451 (PMC7714577; doi:10.1155/2020/7458451)

**Additional Files: Table S1-3**

**Table S1.** Univariable and multivariable Cox regression analysis for all-cause in-hospital mortality.

|  | **Univariable analysis** | | **Multivariable analysis** | |
| --- | --- | --- | --- | --- |
| **Characteristics** | **HR (95% CI)** | ***P* value** | **HR (95% CI)** | ***P* value** |
| **Demographics** |  |  |  |  |
| Age, years | 1.04 (1.03, 1.05) | <0.001 | 1.03 (1.02, 1.04) | <0.001 |
| Sex, male | 0.87 (0.70, 1.09) | 0.230 |  |  |
| BMI, kg/m2 | 0.97 (0.95, 0.99) | <0.001 | 0.99 (0.97, 1.01) | 0.146 |
| **Etiology** |  |  |  |  |
| AMI | 1 |  | 1 |  |
| AHF | 0.22 (0.12, 0.54) | <0.001 | 0.25 (0.16, 0.55) | <0.001 |
| Others | 0.21 (0.09, 0.42) | <0.001 | 0.22 (0.11, 0.48) | <0.001 |
| **History of disease** |  |  |  |  |
| CHF | 0.88 (0.69, 1.12) | 0.296 |  |  |
| AF | 1.19 (0.95, 1.49) | 0.126 |  |  |
| CAD | 1.75 (1.34, 2.91) | 0.001 | 1.68 (1.24, 2.61) | <0.001 |
| Hypertension | 1.13 (0.87, 1.46) | 0.381 |  |  |
| Stroke | 1.87 (1.73, 1.99) | 0.048 | 0.96 (0.74, 1.72) | 0.412 |
| COPD | 1.97 (1.08, 3.60) | 0.028 | 1.85 (0.99, 3.46) | 0.053 |
| DM | 0.89 (0.70, 1.12) | 0.300 |  |  |
| CKD | 1.13 (0.89, 1.42) | 0.316 |  |  |
| Smoke | 0.88 (0.70, 1.09) | 0.234 |  |  |
| **Vital signs at presentation** |  |  |  |  |
| SBP, mmHg | 0.73 (0.59, 0.92) | <0.001 | 0.71 (0.55, 0.88) | <0.001 |
| DBP, mmHg | 1.01 (1.00, 1.01) | 0.303 |  |  |
| HR, beats/min | 1.00 (1.00, 1.01) | 0.651 |  |  |
| SpO2, % | 1.00 (0.99, 1.01) | 0.621 |  |  |
| **Laboratory findings and blood gas analysis** |  |  |  |  |
| NPAR |  |  |  |  |
| Group I | 1 |  | 1 |  |
| Group II | 2.58 (1.90, 3.51) | <0.001 | 2.22 (1.55, 3.16) | <0.001 |
| Group III | 3.74 (2.69, 5.19) | <0.001 | 2.60 (1.72, 3.92) | <0.001 |
| Neutrophil percentage, % | 1.02 (1.01, 1.03) | 0.002 | 0.99 (0.98, 1.00) | 0.155 |
| Albumin, mg/dL | 1.00 (0.82, 1.21) | 0.961 |  |  |
| Creatinine, μmol/L | 1.04 (0.99, 1.11) | 0.143 |  |  |
| Glucose, mg/dL | 1.00 (1.00, 1.00) | 0.194 |  |  |
| BUN, mg/dL | 1.01 (1.00, 1.01) | 0.020 | 1.00 (0.99, 1.00) | 0.460 |
| Hemoglobin, g/dL | 1.07 (0.98, 1.12) | 0.213 |  |  |
| Platelet, 109/L | 1.00 (1.00, 1.00) | 0.079 |  |  |
| WBC, 109/L | 1.00 (0.99, 1.01) | 0.611 |  |  |
| cTnT, ng/mL | 0.95 (0.93, 0.97) | <0.001 | 1.00 (0.97, 1.02) | 0.839 |
| Bicarbonate, mmol/L | 1.26 (1.06, 1.51) | 0.011 | 1.08 (0.89, 1.33) | 0.436 |
| Potassium, mmol/L | 0.99 (0.97, 1.01) | 0.525 |  |  |
| Sodium, mmol/L | 0.99 (0.98, 1.01) | 0.405 |  |  |
| Chloride, mmol/L | 1.04 (1.01, 1.07) | 0.002 | 1.02 (0.99, 1.05) | 0.245 |
| Lactate,mmol/L | 1.07 (1.04, 1.09) | <0.001 | 1.04 (1.01, 1.08) | 0.021 |
| Anion gap, mmol/L | 1.00 (0.98, 1.02) | 0.753 |  |  |
| APTT, second | 1.01 (1.00, 1.01) | 0.047 | 1.00 (0.99, 1.00) | 0.632 |
| PT, second | 1.02 (1.01, 1.03) | 0.002 | 1.02 (0.99, 1.06) | 0.211 |
| INR | 1.14 (1.06, 1.22) | 0.001 | 0.95 (0.74, 1.22) | 0.682 |
| **Scoring system** |  |  |  |  |
| OASIS | 1.05 (1.04, 1.06) | <0.001 | 1.01 (0.99, 1.03) | 0.328 |
| SOFA | 1.08 (1.05, 1.11) | <0.001 | 0.99 (0.94, 1.05) | 0.775 |
| SAPS II | 1.03 (1.02, 1.04) | <0.001 | 1.01 (0.99, 1.02) | 0.359 |
| **In-hospital management** |  |  |  |  |
| Oxygen therapy | 1.49 (1.12, 1.99) | 0.006 | 1.41 (1.02, 1.96) | 0.040 |
| PCI | 0.71 (0.46, 0.88) | <0.001 | 0.80 (0.50, 1.30) | 0.369 |
| CABG | 0.87 (0.66, 1.14) | 0.301 |  |  |
| IABP | 1.64 (0.73, 3.69) | 0.229 |  |  |
| RRT | 1.07 (0.83, 1.38) | 0.592 |  |  |
| In-hospital medication |  |  |  |  |
| Inotrope | 1.00 (0.80, 1.25) | 0.995 |  |  |
| Vasoconstrictor | 1.31 (1.01, 1.70) | 0.039 | 0.79 (0.58, 1.09) | 0.151 |

**Abbreviation**: NPAR: Neutrophil percentage-albumin ratio; HR: Hazard ratio; CI: Confidence interval; BMI: Body mass index; AMI: Acute myocardial infarction; AHF: Acute heart failure; CHF: Chronic heart failure; AF: Atrial fibrillation; CAD: Coronary artery disease, COPD: Chronic obstructive pulmonary disease; DM: Diabetes mellitus; CKD: Chronic kidney disease; SBP: Systolic blood pressure; DBP: Diastolic blood pressure; HR: Heart rate; SpO2: Pulse oximetry-derived oxygen saturation; BUN: Blood urea nitrogen; WBC: White blood cell; cTnT: Cardiac troponin t; APTT: Activated partial thromboplastin time; PT: Prothrombin time; INR: International normalized ratio; OASIS: Oxford Acute Severity of Illness Score; SOFA: Sequential Organ Failure Assessment; SAPS: Simplified Acute Physiology Score; PCI: Percutaneous coronary intervention; CABG: Coronary artery bypass grafting; IABP: Intra-aortic balloon pump; RRT: Renal replacement treatment.

**Table S2.** Univariable and multivariable Cox regression analysis for all-cause 30-day mortality.

|  | **Univariable** | | **Multivariable** | |
| --- | --- | --- | --- | --- |
| **Characteristics** | **HR (95% CI)** | ***P* value** | **HR (95% CI)** | ***P* value** |
| **Demographics** |  |  |  |  |
| Age, years | 1.03 (1.02, 1.04) | <0.001 | 1.02 (1.01, 1.03) | <0.001 |
| Sex, male | 0.92 (0.73, 1.15) | 0.440 |  |  |
| BMI, kg/m^2^ | 0.97 (0.96, 0.99) | 0.001 | 0.99 (0.97, 1.01) | 0.262 |
| **Etiology** |  |  |  |  |
| AMI | 1 |  | 1 |  |
| AHF | 0.20 (0.11, 0.51) | <0.001 | 0.21 (0.14, 0.58) | <0.001 |
| Others | 0.18 (0.08, 0.42) | <0.001 | 0.20 (0.10, 0.45) | <0.001 |
| **History of disease** |  |  |  |  |
| CHF | 1.20 (0.95, 1.53) | 0.128 |  |  |
| AF | 1.33 (1.07, 1.65) | 0.011 | 1.06 (0.83, 1.35) | 0.621 |
| CAD | 1.85 (1.54, 3.21) | <0.001 | 1.78 (1.44, 2.95) | <0.001 |
| Hypertension | 1.13 (0.87, 1.48) | 0.354 |  |  |
| Stroke | 0.56 (0.25, 1.26) | 0.161 |  |  |
| COPD | 1.88 (1.06, 3.36) | 0.031 | 1.64 (0.90, 2.97) | 0.105 |
| DM | 0.92 (0.73, 1.15) | 0.456 |  |  |
| CKD | 1.30 (1.03, 1.64) | 0.031 | 0.97 (0.73, 1.29) | 0.835 |
| Smoke | 0.83 (0.67, 1.03) | 0.088 |  |  |
| **Vital signs at presentation** |  |  |  |  |
| SBP, mmHg | 0.69 (0.45, 0.85) | <0.001 | 0.66 (0.40, 0.81) | <0.001 |
| DBP, mmHg | 1.01 (1.00, 1.02) | 0.072 |  |  |
| HR, beats/min | 1.00 (1.00, 1.01) | 0.789 |  |  |
| SpO_2_, % | 1.00 (0.99, 1.01) | 0.695 |  |  |
| **Laboratory findings and blood gas analysis** |  |  |  |  |
| NPAR |  |  |  |  |
| Group I | 1 |  | 1 |  |
| Group II | 2.34 (1.76, 3.11) | <0.001 | 1.96 (1.42, 2.71) | <0.001 |
| Group III | 3.69 (2.70, 5.05) | <0.001 | 2.42 (1.65, 3.54) | <0.001 |
| Neutrophil percentage, % | 1.01 (1.00, 1.02) | 0.026 | 0.99 (0.98, 1.01) | 0.274 |
| Albumin, mg/dL | 0.85 (0.71, 1.03) | 0.099 |  |  |
| Creatinine, μmol/L | 1.08 (1.02, 1.14) | 0.007 | 0.95 (0.85, 1.06) | 0.400 |
| Glucose, mg/dL | 1.00 (1.00, 1.00) | 0.302 |  |  |
| BUN, mg/dL | 1.01 (1.01, 1.01) | <0.001 | 1.00 (1.00, 1.01) | 0.450 |
| Hemoglobin, g/dL | 1.01 (0.96, 1.06) | 0.780 |  |  |
| Platelet, 10^9^/L | 1.00 (1.00, 1.00) | 0.149 |  |  |
| WBC, 10^9^/L | 1.00 (1.00, 1.01) | 0.350 |  |  |
| cTnT, ng/mL | 0.95 (0.93, 0.97) | <0.001 | 1.01 (0.99, 1.04) | 0.359 |
| Bicarbonate, mmol/L | 1.34 (1.12, 1.60) | 0.002 | 1.07 (0.88, 1.29) | 0.504 |
| Potassium, mmol/L | 0.99 (0.97, 1.02) | 0.581 |  |  |
| Sodium, mmol/L | 0.99 (0.97, 1.00) | 0.114 |  |  |
| Chloride, mmol/L | 1.06 (1.04, 1.09) | <0.001 | 1.02 (0.99, 1.05) | 0.250 |
| Lactate,mmol/L | 1.10 (1.08, 1.13) | <0.001 | 1.07 (1.03, 1.11) | <0.001 |
| Anion gap, mmol/L | 1.01 (0.99, 1.03) | 0.654 |  |  |
| APTT, second | 1.01 (1.00, 1.01) | 0.012 | 1.00 (1.00, 1.01) | 0.484 |
| PT, second | 1.02 (1.01, 1.03) | 0.001 | 1.01 (0.98, 1.05) | 0.376 |
| INR | 1.13 (1.06, 1.21) | <0.001 | 0.98 (0.78, 1.22) | 0.831 |
| **Scoring system** |  |  |  |  |
| OASIS | 1.05 (1.04, 1.07) | <0.001 | 1.01 (0.99, 1.03) | 0.293 |
| SOFA | 1.12 (1.08, 1.15) | <0.001 | 1.02 (0.97, 1.08) | 0.399 |
| SAPS II | 1.04 (1.03, 1.04) | <0.001 | 1.01 (0.99, 1.03) | 0.195 |
| **In-hospital management** |  |  |  |  |
| Oxygen therapy | 1.39 (1.06, 1.83) | 0.017 | 1.22 (0.89, 1.68) | 0.219 |
| PCI | 0.74 (0.57, 0.98) | 0.032 | 0.80 (0.59, 1.10) | 0.185 |
| CABG | 0.44 (0.29, 0.67) | <0.001 | 0.70 (0.44, 1.11) | 0.129 |
| IABP | 0.89 (0.28, 2.76) | 0.836 |  |  |
| RRT | 1.48 (1.15, 1.90) | 0.002 | 0.91 (0.68, 1.23) | 0.551 |
| In-hospital medication |  |  |  |  |
| Inotrope | 1.16 (0.93, 1.45) | 0.181 |  |  |
| Vasoconstrictor | 1.36 (1.05, 1.76) | 0.020 | 0.74 (0.54, 1.02) | 0.067 |

**Abbreviation**: NPAR: Neutrophil percentage-albumin ratio; HR: Hazard ratio; CI: Confidence interval; BMI: Body mass index; AMI: Acute myocardial infarction; AHF: Acute heart failure; CHF: Chronic heart failure; AF: Atrial fibrillation; CAD: Coronary artery disease, COPD: Chronic obstructive pulmonary disease; DM: Diabetes mellitus; CKD: Chronic kidney disease; SBP: Systolic blood pressure; DBP: Diastolic blood pressure; HR: Heart rate; SpO2: Pulse oximetry-derived oxygen saturation; BUN: Blood urea nitrogen; WBC: White blood cell; cTnT: Cardiac troponin t; APTT: Activated partial thromboplastin time; PT: Prothrombin time; INR: International normalized ratio; OASIS: Oxford Acute Severity of Illness Score; SOFA: Sequential Organ Failure Assessment; SAPS: Simplified Acute Physiology Score; PCI: Percutaneous coronary intervention; CABG: Coronary artery bypass grafting; IABP: Intra-aortic balloon pump; RRT: Renal replacement treatment.

**Table S3.** Univariable and multivariable Cox regression analysis for all-cause 365-day mortality.

|  | **Univariable** | | **Multivariable** | |
| --- | --- | --- | --- | --- |
| **Characteristics** | **HR (95% CI)** | ***P* value** | **HR (95% CI)** | ***P* value** |
| **Demographics** |  |  |  |  |
| Age, years | 1.03 (1.02, 1.04) | <0.001 | 1.02 (1.01, 1.03) | <0.001 |
| Sex, male | 0.97 (0.81, 1.16) | 0.727 |  |  |
| BMI, kg/m^2^ | 0.98 (0.97, 0.99) | 0.005 | 0.99 (0.98, 1.01) | 0.465 |
| **Etiology** |  |  |  |  |
| AMI | 1 |  | 1 |  |
| AHF | 0.24 (0.15, 0.61) | <0.001 | 0.23 (0.12, 0.57) | <0.001 |
| Others | 0.18 (0.05, 0.45) | <0.001 | 0.19 (0.08, 0.47) | <0.001 |
| **History of disease** |  |  |  |  |
| CHF | 1.37 (1.13, 1.67) | 0.001 | 1.06 (0.86, 1.30) | 0.597 |
| AF | 1.26 (1.06, 1.51) | 0.011 | 1.05 (0.86, 1.28) | 0.643 |
| CAD | 0.88 (0.74, 1.06) | 0.177 |  |  |
| Hypertension | 1.27 (1.02, 1.57) | 0.030 | 0.86 (0.61, 1.22) | 0.395 |
| Stroke | 0.66 (0.36, 1.20) | 0.177 |  |  |
| COPD | 1.72 (1.05, 2.84) | 0.032 | 1.41 (0.84, 2.36) | 0.195 |
| DM | 0.95 (0.79, 1.14) | 0.582 |  |  |
| CKD | 1.42 (1.18, 1.72) | <0.001 | 1.24 (0.90, 1.71) | 0.194 |
| Smoke | 0.87 (0.73, 1.04) | 0.127 |  |  |
| **Vital signs at presentation** |  |  |  |  |
| SBP, mmHg | 0.78 (0.64, 0.95) | <0.001 | 0.80 (0.67, 0.98) | 0.004 |
| DBP, mmHg | 1.01 (1,00, 1.01) | 0.112 |  |  |
| HR, beats/min | 1.00 (1.00, 1.01) | 0.169 |  |  |
| SpO_2_, % | 1.00 (1.00, 1.01) | 0.433 |  |  |
| **Laboratory findings and blood gas analysis** |  |  |  |  |
| NPAR |  |  |  |  |
| Group I | 1 |  | 1 |  |
| Group II | 2.00 (1.61, 2.50) | <0.001 | 3.03 (2.14, 4.28) | <0.001 |
| Group III | 3.43 (2.67, 4.41) | <0.001 | 6.80 (4.10, 11.26) | <0.001 |
| Neutrophil percentage, % | 1.01 (1.01, 1.02) | 0.002 | 2.09 (1.59, 2.75) | <0.001 |
| Albumin, mg/dL | 0.71 (0.61, 0.84) | <0.001 | 0.98 (0.97, 0.99) | <0.001 |
| Creatinine, μmol/L | 1.08 (1.03, 1.13) | <0.001 | 0.94 (0.85, 1.03) | 0.165 |
| Glucose, mg/dL | 1.00 (1.00, 1.00) | 0.339 |  |  |
| BUN, mg/dL | 1.01 (1.01, 1.01) | <0.001 | 1.00 (1.00, 1.01) | 0.157 |
| Hemoglobin, g/dL | 0.96 (0.92, 1.00) | 0.051 |  |  |
| Platelet, 10^9^/L | 1.00 (1.00, 1.00) | 0.090 |  |  |
| WBC, 10^9^/L | 1.00 (1.00, 1.01) | 0.195 |  |  |
| cTnT, ng/mL | 0.97 (0.95, 0.99) | <0.001 | 1.02 (1.00, 1.04) | 0.066 |
| Bicarbonate, mmol/L | 1.28 (1.11, 1.49) | 0.001 | 1.07 (0.91, 1.26) | 0.385 |
| Potassium, mmol/L | 1.00 (0.98, 1.02) | 0.863 |  |  |
| Sodium, mmol/L | 0.99 (0.98, 1.00) | 0.119 |  |  |
| Chloride, mmol/L | 1.06 (1.03, 1.08) | <0.001 | 1.04 (1.01, 1.07) | 0.006 |
| Lactate,mmol/L | 1.09 (1.07, 1.12) | <0.001 | 1.05 (1.02, 1.08) | 0.001 |
| Anion gap, mmol/L | 1.01 (0.99, 1.02) | 0.496 |  |  |
| APTT, second | 1.01 (1.00, 1.01) | 0.002 | 1.00 (1.00, 1.01) | 0.041 |
| PT, second | 1.02 (1.01, 1.02) | <0.001 | 1.00 (0.98, 1.04) | 0.749 |
| INR | 1.12 (1.06, 1.18) | <0.001 | 1.04 (0.84, 1.27) | 0.731 |
| **Scoring system** |  |  |  |  |
| OASIS | 1.05 (1.04, 1.06) | <0.001 | 1.01 (1.00, 1.03) | 0.098 |
| SOFA | 1.10 (1.08, 1.13) | <0.001 | 1.00 (0.95, 1.04) | 0.874 |
| SAPS II | 1.03 (1.03, 1.04) | <0.001 | 1.01 (1.00, 1.02) | 0.090 |
| **Management of AHF** |  |  |  |  |
| Oxygen therapy | 1.31 (1.05, 1.63) | 0.015 | 1.22 (0.94, 1.59) | 0.135 |
| PCI | 0.70 (0.44, 0.85) | 0.002 | 0.81 (0.62, 1.15) | 0.110 |
| CABG | 0.46 (0.33, 0.64) | <0.001 | 0.66 (0.46, 0.94) | 0.021 |
| IABP | 1.67 (0.79, 3.52) | 0.181 |  |  |
| RRT | 1.68 (1.37, 2.06) | <0.001 | 1.17 (0.91, 1.49) | 0.224 |
| In-hospital medication |  |  |  |  |
| Inotrope | 1.16 (0.97, 1.39) | 0.112 |  |  |
| Vasoconstrictor | 1.37 (1.11, 1.69) | 0.004 | 0.91 (0.70, 1.19) | 0.498 |

**Abbreviation**: NPAR: Neutrophil percentage-albumin ratio; HR: Hazard ratio; CI: Confidence interval; BMI: Body mass index; AMI: Acute myocardial infarction; AHF: Acute heart failure; CHF: Chronic heart failure; AF: Atrial fibrillation; CAD: Coronary artery disease, COPD: Chronic obstructive pulmonary disease; DM: Diabetes mellitus; CKD: Chronic kidney disease; SBP: Systolic blood pressure; DBP: Diastolic blood pressure; HR: Heart rate; SpO2: Pulse oximetry-derived oxygen saturation; BUN: Blood urea nitrogen; WBC: White blood cell; cTnT: Cardiac troponin t; APTT: Activated partial thromboplastin time; PT: Prothrombin time; INR: International normalized ratio; OASIS: Oxford Acute Severity of Illness Score; SOFA: Sequential Organ Failure Assessment; SAPS: Simplified Acute Physiology Score; PCI: Percutaneous coronary intervention; CABG: Coronary artery bypass grafting; IABP: Intra-aortic balloon pump; RRT: Renal replacement treatment.

**Additional file: Figure S1**

**Figure legends**

Fig S1. Receiver operating characteristic curve of neutrophil percentage, albumin and NPAR values. (A) and (B): in-hospital mortality. (C) and (D): 30-day mortality. (E) and (F): 365-day mortality. Abbreviations: AUC: Area under the curve; NPAR: Neutrophil percentage-albumin ratio.


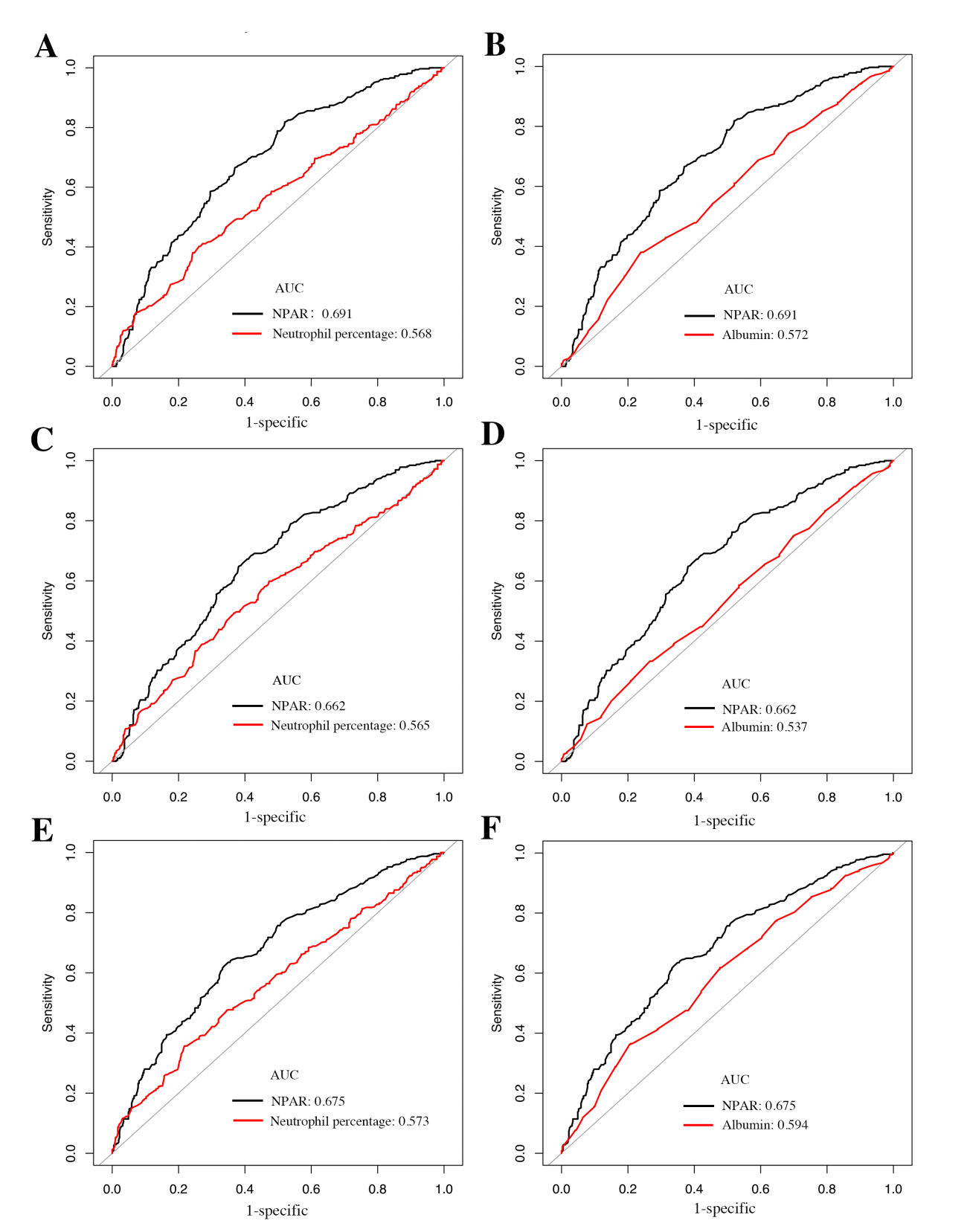

Supplement: Supplementary Materials — Table S1: univariable and multivariable Cox regression analysis for all-cause in-hospital mortality. Table S2: univariable and multivariable Cox regression analysis for all-cause 30-day mortality. Table S3: univariable and multivariable Cox regression analysis for all-cause 365-day mortality. Figure S1: receiver operating characteristic curve of the neutrophil percentage, albumin, and NPAR values. (a, b) in-hospital mortality. (c, d) 30-day mortality. (e, f) 365-day mortality. Abbreviations: AUC: area under the curve; NPAR: neutrophil percentage-albumin ratio. [file 7458451.f1.docx]
